# Rates and characteristics of suicide by immigration background in Norway

**DOI:** 10.1371/journal.pone.0205035

**Published:** 2018-09-28

**Authors:** Quirino Puzo, Lars Mehlum, Ping Qin

**Affiliations:** National Centre for Suicide Research and Prevention, Institute of Clinical Medicine, University of Oslo, Oslo, Norway; Karolinska Institutet, SWEDEN

## Abstract

Suicide mortality among immigrant groups is an important health issue, particularly in countries with growing segments of immigrant populations such as Norway. Through linkage of Norwegian national registers we wanted to estimate suicide rates (per 100,000 population) in immigrant groups and to profile characteristics of suicide by immigration background with respect to sex, age, method and seasonality of suicide as well as time since immigrating to Norway. Among all 11,409 suicides during 1992–2012, 1,139 (10%) were individuals with an immigration background. Suicide rate was lower in first-generation immigrants (foreign-born persons to two foreign-born parents) than native Norwegians (9.53 vs 12.22, *P* < 0.01), with a significant difference confined to male rates only. Foreign-born persons with at least one Norwegian-born parent had significantly higher suicide rates than natives in both sexes (22.42 vs 18.03 in males, 11.67 vs 6.54 in females, *P* < 0.01). The most frequently used suicide method in all the population groups was hanging; this method accounted for 44.0% of all suicides of first-generation immigrants, 45.2% of all suicides of foreign-born persons with at least one Norwegian-born parent, and 35.4% of all suicides of natives. Suicide by firearms accounted for a much smaller proportion of cases of first-generation immigrants (6.7%) and foreign-born persons with at least one Norwegian-born parent (6.8%) than cases of native Norwegians (20.7%). In terms of monthly distribution, suicides of first-generation immigrants displayed two peaks, in May and in November (*P* = 0.01). More than 25% of all first-generation immigrant suicides occurred in the first five years after immigration; but differences in time since immigration were observed by sex and country group of origin, in particular among those aged 35 years or less when moving to Norway. In conclusion, there are notable differences in characteristics of suicides by immigration background. Knowledge of immigrant mortality according to suicide method, seasonality of suicide, and time since immigration may be useful when planning suicide preventive measures.

## Introduction

During the last decades the number of immigrants to European countries has grown dramatically, as a result of societal phenomena such as increasing labour demands in the growing economies, family reunifications and refugee streams due to wars and political conflicts [1]. According to the UN, the estimated number of international immigrants (people born outside of the country they live in) in Europe in 2017 was almost 78 million, representing approximately 10.5% of the entire European population [2]. During the process integrating to the host country, immigrants may experience multiple stresses that may impact their mental wellbeing and may increase risks of mental disorders and suicidal behaviour [3–7]. Cultures of origin, the relative social and economic marginalization of immigrant minority groups from the majority society, and the weakening of previous family and social networks are suggested factors that influence immigrants’ suicidal behaviour [5, 8–10]. Studies comparing immigrants’ suicide rates with those of the native population have reported divergent results. Most studies have shown that immigrants tend to retain suicide rates of their country of origin while residing in the host countries [11–13], while some studies have demonstrated that, with increasing time spent in the immigration countries, suicide rates of immigrant groups appear to converge with the rates of the native population in the host country [12, 14]. Other studies have shown that foreign-born persons (persons born in a country other than the one in which they reside) have a significantly higher rate of suicide death compared with the indigenous population [15, 16].

Norway is among the countries where the proportion of first-generation immigrants (foreign-born persons to two foreign-born parents) and second-generation immigrants (Norwegian-born persons to two foreign-born parents) in the population has increased significantly, from around 1.5% of the national population in the early 1970s to 16.8% in 2017, with a total of 883,751 persons [17]. In a previous population study we have demonstrated that first- and second-generation immigrants had a significantly lower risk of suicide compared with native Norwegians, whereas the risks for Norwegian-born persons with one foreign-born parent and foreign-born individuals with at least one Norwegian-born parent were significantly higher [18]. We have furthermore shown that various immigrant populations, as well as native Norwegians, share most common risk factors for suicide such as low levels of education, low annual income and single, separated, divorced or widowed marital status; whereas living in the capital area was associated with a reduced risk of suicide in first-generation immigrants but an increased risk in native Norwegians [19].

Despite these findings, there is a lack of knowledge on absolute rates of suicide by immigration background and of research into detailed characteristics of suicides by this background. Understanding differences in characteristics of suicides by specific immigration background is of fundamental importance especially for professionals, who, aware of such differences while planning suicide preventive measure, may more effectively tailor these measures to at-risk immigrant populations. Using the unique possibilities of Norwegian national registers, the aims of this study were: (1) to estimate rates of suicide death for immigrant groups and the majority population, (2) to profile characteristics of suicides by immigration background with respect to sex, age, method of suicide, and month of the year when suicide occurred and (3) to investigate temporal patterns of suicide incidents of first-generation immigrants according to time since their immigration to Norway.

## Materials and methods

### Data sources, study design and population

Data for the present study were retrieved from two Norwegian national longitudinal registers that were interlinked on an individual level via the encrypted personal identifier of all residents in Norway. The Cause-of-Death Register contains dates and causes of all deaths in Norway since the year 1969. Suicide was coded according to the International Classification of Diseases (ICD), 9th Revision for the years 1992–95 (codes E950-E959) and 10th Revision for the years 1996–2012 (codes X60-X84, Y870). The Central Population Register, computerized since the year 1964, contains demographic data such as country of birth, citizenship, date of immigration or emigration, alongside with sex, date of birth and links to parents.

In this retrospective population-based study, cases were identified as all completed suicides from the Cause-of-Death Register from the year 1992 through 2012 and were restricted to individuals residing in Norway at the time of their suicide, yielding a total of 11,409 suicide cases.

### Variables

#### Immigration background

Data on immigration background for suicide cases were obtained from the Central Population Register, and categorized, considering the Statistics Norway’s classification on immigration background [20], into the following five groups: native Norwegians (Norwegian-born persons to two Norwegian-born parents), first-generation immigrants (foreign-born persons to two foreign-born parents), second-generation immigrants (Norwegian-born persons to two foreign-born parents), Norwegian-born persons with one foreign-born parent, and foreign-born individuals with at least one Norwegian-born parent (including intercountry adoptees, for which our data sources do not allow us to distinguish). The categorization was made upon available information of legal parents since the establishment of Central population register in 1964 [21].

#### Method of suicide

Data on method of suicide were retrieved from the Cause-of-Death Register for the period under study and classified into seven categories: “Poisoning” (E950-E952 or X60-X69), “Hanging” (E953 or X70), “Drowning” (E954 or X71), “Firearms and explosive material” (E955 or X72-X75), “Cutting or piercing instruments” (E956 or X78), “Jumping from a high place” (E957 or X80) and “Other or unknown method” (E958-E959 or X76-77, X79, X81-84, Y870). The category “Firearms and explosive material” was relabelled as “Firearms” in the text since suicides in this group almost exclusively contained suicides by firearms.

#### Month of suicide

Month of suicide refers to the month of the year when the suicide occurred.

#### Time since immigration

Time since immigration refers to the time span between the date of immigration to Norway (i.e., registered as a resident in the population registration system) and the date of suicide death, and it was classified with yearly increments (up to 1 year, 1-up to 2 years, 2-up to 3 years, etc.). This variable applies only to first-generation immigrants, who were further divided into seven geographically defined country groups of origin (Nordic countries, Western Europe except Nordic countries, Eastern Europe, Asia including Turkey, Africa, North America and Oceania, Central and South America).

### Statistical analysis

Rates of suicide for various immigration groups by sex were calculated for the period 1992–2012 using the population distribution by immigration background available on the Statistics Norway website [22, 23]. Suicide rates were expressed as number per 100,000 population. Differences in suicide mortality rates among various immigrant groups were tested using T-test for independent 2-groups.

Absolute numbers and proportions of suicides by immigration background with respect to sex, age groups, method of suicide, and month of suicide were tabulated. Chi-squared test or Fisher’s exact test was conducted to assess differences in categorical variables between the studied groups. Chi-square goodness of fit test was used to verify whether the observed distribution of suicides in the studied groups was equal on the time axis (month of the year). Mann-Whitney test was performed to assess differences in time since immigration among first-generation immigrants by sex and by country group of origin. Data were analysed using R statistical software (version 3.3.3) [24]. For all the statistical tests, a *P* value smaller than 5% was considered to be statistically significant.

### Ethical considerations

Access to data for the study was approved by the Regional Ethical Committee for Medical and Health Research (REK sør-øst) and owners of the relevant individual registers. No informed consent from the participants was obtained because this was a population-based study with data from national registers.

## Results

A total of 11,409 suicides were registered between 1992 and 2012 in Norway, of which 8,277 were males (72.5%) and 3,132 were females (27.5%). Their ages ranged from 8 to 100 years, with a median age of 44 years (SD = 18.52); 43 years (SD = 18.71) for males and 46 years (SD = 17.96) for females. Individuals with an immigration background comprised 10.0% (N = 1,139) of all suicides; 5.0% (N = 566) were individuals born abroad to two foreign-born parents (first-generation immigrants), 0.3% (N = 30) were born in Norway to two foreign-born parents (second-generation immigrants), 3.2% (N = 366) were born in Norway with one foreign-born parent, and 1.6% (N = 177) were born abroad with at least one Norwegian-born parent.

### Suicide mortality by immigration background

Considering both sexes, the suicide mortality rate (per 100,000 population) was 12.22 for native Norwegians, 9.53 for first-generation immigrants, 2.56 for second-generation immigrants, 11.13 for Norwegian-born with one foreign-born parent, and 17.10 for foreign-born with at least one Norwegian-born parent (Table 1).

**Table 1:** Suicide rate (per 100,000 population) by immigration background, 1992–2012.

	Average annual suicide rate^a^ (95% CI)			*M*:*F ratio*
*Immigration Background*	Both sexes	Males	Females	
Native Norwegians	12.22 (11.8–12.6)	18.03 (17.3–18.8)	6.54 (6.3–6.8)	2.76
First-generation immigrants	9.53 (8.7–10.4)	12.73 (11.5–14.0)	6.29 (5.5–7.1)	2.02
Second-generation immigrants	2.56 (1.5–3.6)	3.99 (1.9–6.1)	1.05 (0.2–1.9)	3.80
Norwegian-born with one foreign-born parent	11.13 (9.7–12.6)	15.70 (13.6–17.8)	6.21 (4.7–7.7)	2.53
Foreign-born with at least one Norwegian-born parent	17.10 (14.6–19.6)	22.42 (18.9–25.9)	11.67 (8.3–15.0)	1.92

^a^ Suicide rates were not age-standardized because the age distribution of all immigrant populations was not available.

The suicide rate of first-generation immigrants was significantly lower than that of native Norwegians (9.53 vs 12.22, *P* < 0.01). This difference remained statistically significant only among males (12.73 vs 18.03, *P* < 0.01) and not among females (6.29 vs 6.54, *P* = 0.56).

Second-generation immigrants showed very low suicide rates in both sexes (3.99 in males and 1.05 in females), but these estimates should be interpreted with caution because second-generation immigrants represent a relatively younger group of people in this society (see Discussion).

No statistically significant differences were observed in suicide rates between Norwegian-born with one foreign-born parent and native Norwegians (15.70 vs 18.03 in males, *P* = 0.06; 6.21 vs 6.54 in females, *P* = 0.67). Foreign-born persons with at least one Norwegian-born parent had, however, significantly higher suicide rates than the natives, for both sexes (22.42 vs 18.03 in males, *P* < 0.01; 11.67 vs 6.54 in females, *P* < 0.01). The male to female ratio (M:F) was somewhat lower among first-generation immigrants and foreign-born with at least one Norwegian born-parent as compared to that of native Norwegians.

### Characteristics of suicides by immigration background

The comparison between native Norwegians and the immigrant groups showed overall significant differences in the distribution of suicides by sex, age and method of suicide (Table 2). More than 45% of suicide cases of first-generation immigrants were aged 25–44, while the proportion of native Norwegians who died by suicide at this age was 35.0%. The same pattern was found in the other immigrant groups where suicide cases aged 25–44 constituted more than 42% of all suicides. Clearly, the median age of suicide cases with an immigration background was much lower than that of suicides of the natives; 42 years, 27 years, 32 years, 30 years and 45 years respectively for first-generation immigrants, second-generation immigrants, Norwegian-born with one foreign-born parent, foreign-born with at least one Norwegian-born parent, and native Norwegians.

**Table 2:** Characteristics of suicide cases by immigration background with respect to sex, age, method of suicide, and month of suicide, 1992–2012.

	Native Norwegians	First-generation immigrants	Second-generation immigrants	Norwegian-born with one foreign-born parent	Foreign-born with at least one Norwegian-born parent	*P-value*
	(N = 10,270)	(N = 566)	(N = 30)	(N = 366)	(N = 177)	
	*N (%)*	*N (%)*	*N (%)*	*N (%)*	*N (%)*	
**Sex**						
Males	7,492 (73.0)	381 (67.3)	23 (76.7)	268 (73.2)	113 (63.8)	P < 0.01
Females	2,778 (27.0)	185 (32.7)	7 (23.3)	98 (26.8)	64 (36.2)	
**Age**						
<25	1,502 (14.6)	64 (11.3)	13 (43.3)	106 (29.0)	61 (34.5)	P < 0.01
25–44	3,591 (35.0)	257 (45.4)	13 (43.3)	157 (42.9)	87 (49.2)	
45–64	2,629 (25.6)	151 (26.7)	4 (13.3)	70 (19.1)	22 (12.4)	
> = 65	2,548 (24.8)	94 (16.6)	0 (0.0)	33 (9.0)	7 (4.0)	
**Method of suicide**						
Hanging	3,632 (35.4)	249 (44.0)	13 (43.3)	142 (38.8)	80 (45.2)	P < 0.01
Poisoning	2,380 (23.2)	128 (22.6)	3 (10.0)	97(26.5)	45 (25.4)	
Firearms	2,129 (20.7)	38 (6.7)	5 (16.7)	57 (15.6)	12 (6.8)	
Drowning	735 (7.2)	45 (8.0)	0 (0.0)	16 (4.4)	10 (5.6)	
Jumping from a high place	487 (4.7)	44 (7.8)	3 (10.0)	25 (6.8)	14 (7.9)	
Cutting or piercing instruments	254 (2.5)	14 (2.5)	2 (6.7)	4 (1.1)	4 (2.3)	
Other or unknown method	653 (6.4)	48 (8.5)	4 (13.3)	25 (6.8)	12 (6.8)	
**Month of suicide**						
January	871 (8.5)	41 (7.2)	1 (3.3)	25 (6.8)	17 (9.6)	P = 0.09^a^
February	829 (8.1)	36 (6.4)	4 (13.3)	36 (9.8)	12 (6.8)	
March	895 (8.7)	42 (7.4)	3 (10)	33 (9.0)	14 (7.9)	
April	913 (8.9)	34 (6.0)	3 (10)	43 (11.7)	15 (8.5)	
May	946 (9.2)	66 (11.7)	3 (10)	30 (8.2)	15 (8.5)	
June	857 (8.3)	45 (8.0)	4 (13.3)	24 (6.6)	11 (6.2)	
July	809 (7.9)	57 (10.1)	2 (6.7)	35 (9.6)	9 (5.1)	
August	890 (8.7)	48 (8.5)	2 (6.7)	31 (8.5)	18 (10.2)	
September	828 (8.1)	46 (8.1)	0 (0.0)	27 (7.4)	12 (6.8)	
October	856 (8.3)	38 (6.7)	1 (3.3)	25 (6.8)	16 (9.0)	
November	786 (7.7)	63 (11.1)	4 (13.3)	26 (7.1)	21 (11.9)	
December	790 (7.7)	50 (8.8)	3 (10.0)	31 (8.5)	17 (9.6)	

^a^ Due to small numbers of suicides of second-generation immigrants by month, the test was made by comparing the natives with those with immigration background combined.

Hanging was the most common suicide method during the observation period across all population groups under study; this method accounted for 44.0% of all suicides of first-generation immigrants, for 45.2% of all suicides of foreign-born persons with at least one Norwegian-born parent, and for 35.4% of all suicides of natives. Additional analysis of method-specific suicide mortality showed hanging-specific suicide rates (per 100,000 population) of 4.06, 7.56 and 4.32 for first-generation immigrants, foreign-born persons with at least one Norwegian-born parent, and natives, respectively. Suicide by firearms accounted for a much smaller proportion of cases among first-generation immigrants (6.7%) and foreign-born persons with at least one Norwegian-born parent (6.8%) than among native Norwegians (20.7%). In males in particular, firearms accounted for 9.7%, 10.6% and 27.6% in first-generation immigrants, foreign-born persons with at least one Norwegian-born parent, and natives, corresponding to a firearm-specific suicide rate of 1.24, 2.60 and 4.98 respectively.

**Fig 1:**
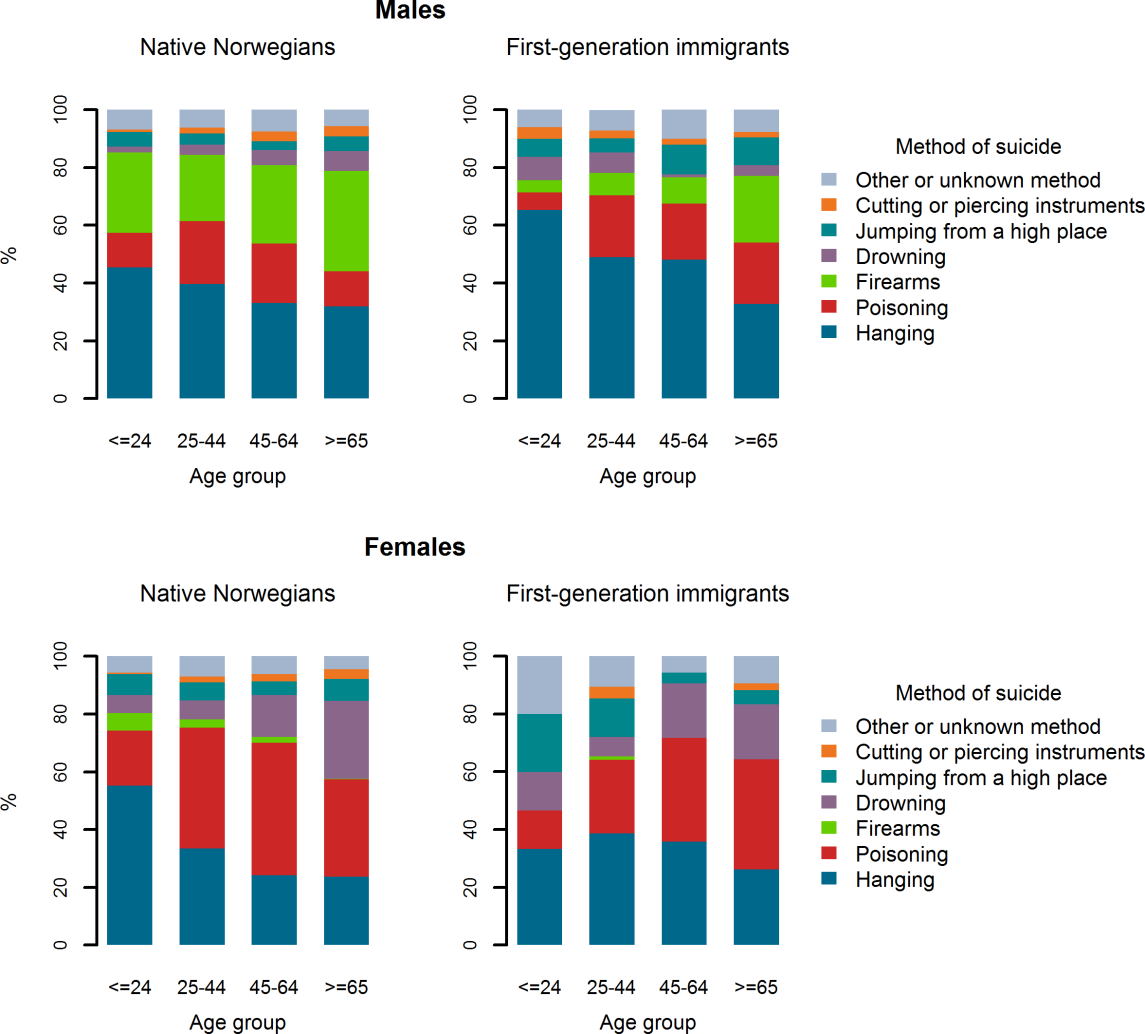
Distribution of suicide cases by specific methods in various age groups of native Norwegians and first-generation immigrants, 1992–2012.

Fig 1 displays the age distribution of method-specific suicides of the first-generation immigrants and the natives. Among male first-generation immigrants, younger individuals (24 years old or less) who died by firearms represented 4.1% of suicides in this age group; the corresponding percentage among male native Norwegians was around 28%. In both natives and first-generation immigrants, females aged 45 years or more mostly died by poisoning, whereas younger females (24 years old or less) more frequently used the method of hanging to take their own life.

**Fig 2:**
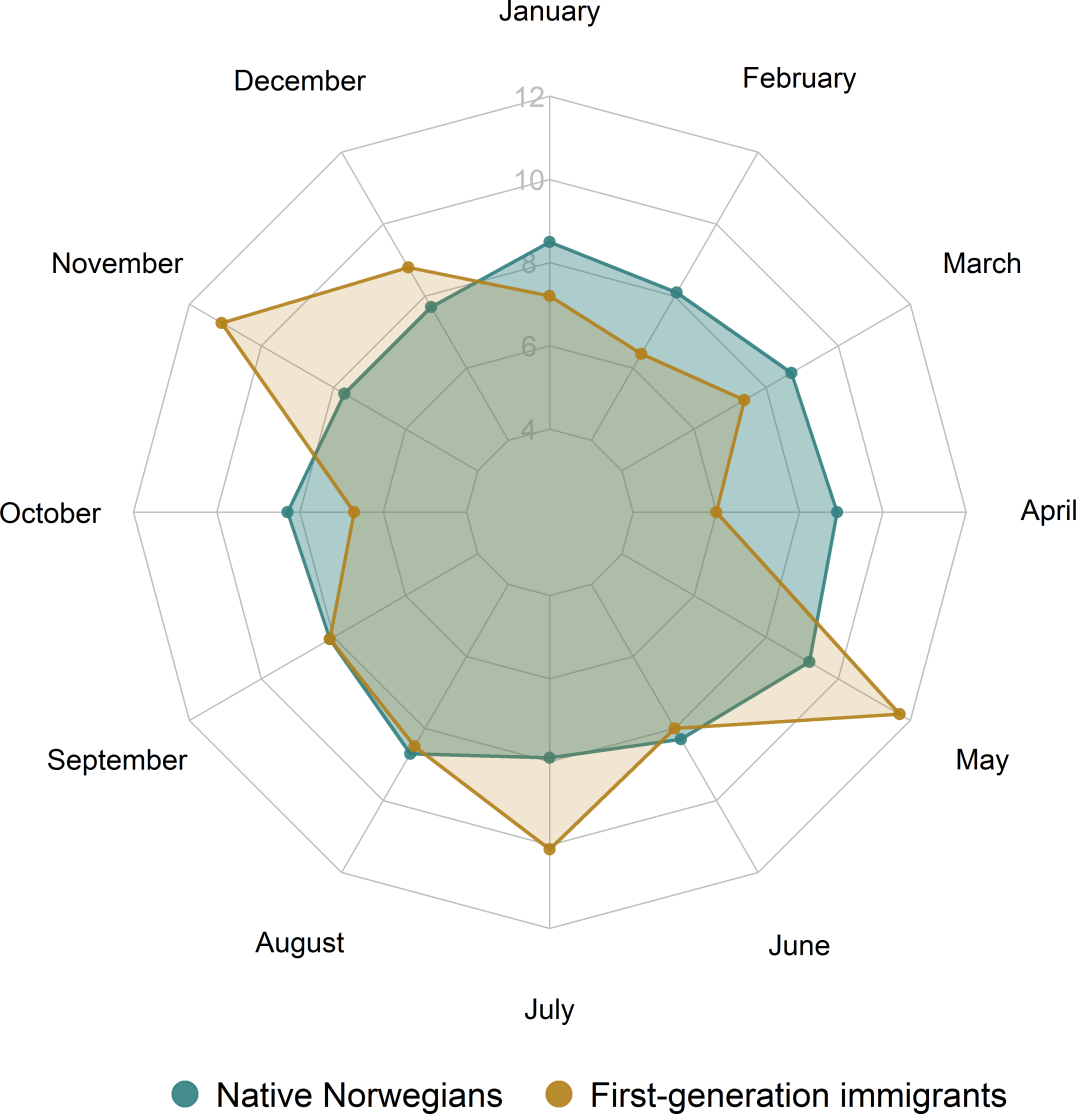
Monthly distribution of suicides of the natives and first-generation immigrants, 1992–2012 (%). Distribution of suicides of both natives and first-generation immigrants demonstrate a significant difference from the hypothesized equal distribution (*P* < 0.01 and *P* = 0.01, respectively).

The overall distribution of suicides by month of the year exhibited a marginal statistical difference between natives and people with immigration background combined (*P* = 0.09), but we observed different patterns of suicide occurrence by month comparing specifically natives and first-generation immigrants (*P* < 0.01). As shown in Fig 2, suicides of native Norwegians demonstrated a tendency of an increased occurrence in the spring (*P* < 0.01), with the highest level seen in May (9.2% of all cases), whereas suicides of first-generation immigrants displayed two peaks in their incidence, in May and in November (11.7% and 11.1% of all cases, respectively) (*P* = 0.01).

### Time since immigration

**Fig 3:**
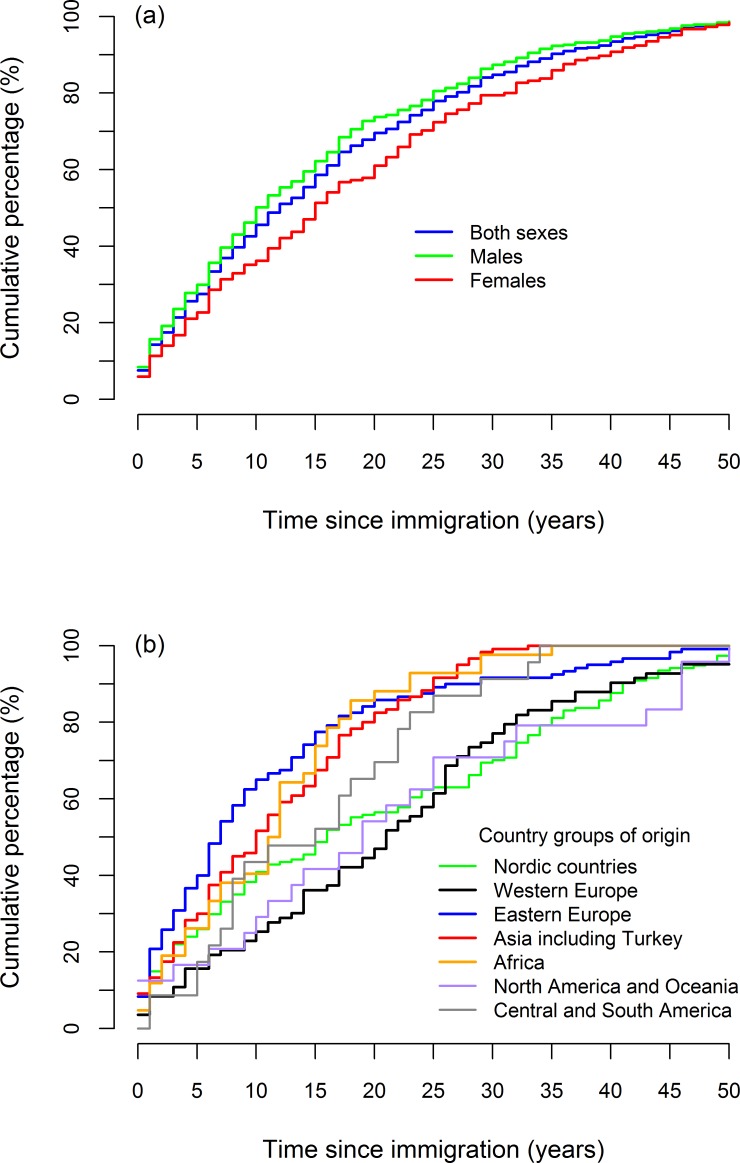
Cumulative percentage distribution of suicides of first-generation immigrants by sex (a) and country group of origin (b) according to time since immigration^a^. ^a^Time since immigration refers to the time span between the date of immigration to Norway and the date of suicide death.

Of the 566 first-generation immigrants who died by suicide during the period 1992–2012, 27.2% (N = 154) were from Nordic countries, 14.7% (N = 83) from Western Europe except Nordic countries, 21.2% (N = 120) from Eastern Europe, 21.2% (N = 120) from Asia including Turkey, 7.4% (N = 42) from Africa, 4.2% (N = 24) from North America and Oceania, and 4.1% (N = 23) from Central and South America. Fig 3 shows the cumulative percentage distribution of suicides of first-generation immigrants by sex and country group of origin according to time since immigration.

More than 25% of suicides of first-generation immigrants occurred within the first five years after immigrating to Norway. Overall, suicides of male first-generation immigrants tended to occur somewhat sooner than that of female first-generation immigrants after arriving in Norway (*P* < 0.01; median time to suicide since immigration: 10.85 years for males and 15.48 years for females). Moreover, differences in time since immigration were observed among first-generation immigrants by country group of origin. Immigrants from Eastern Europe, Asia and Africa died by suicide significantly earlier after immigration than immigrants from Western Europe (*P* < 0.01, *P* < 0.01 and *P* < 0.01, respectively). The median time to suicide since immigration was 7.26 years for Eastern European immigrants, 10.51 years for Asian immigrants, 11.79 years for African immigrants and 21.45 years for immigrants from Western Europe. The observed differences in temporal occurrences of suicides of first-generation immigrants by sex and country group of origin according to time since immigration were confined to young first-generation immigrants (aged 35 years or less) only.

## Discussion

To the best of our knowledge, this is the first population register-based study to provide information on characteristics of suicides (rates, suicide methods, suicide occurrence by month of the year, and time since immigration) using a very detailed classification of persons’ immigration backgrounds.

### Suicide rates

The finding that the suicide rate of first-generation immigrants was comparatively lower than the suicide rate of native Norwegians is in line with what we previously found when studying the relative risk of suicide by immigration background [18]. This may be partly explained by a general improvement of life quality attained by most immigrants in Norway compared to what they had in their country of origin, and the healthy migrant effect, suggesting that first-generation immigrants tend to represent a particularly healthy segment of the population in the country of origin [25–28]. Moreover, since a substantial proportion of first-generation immigrants originates from non-Western countries such as Iraq, Syria, Pakistan, Iran and the Philippines [29] with relatively low suicide rates [30], this suggests that first-generation immigrants tend to retain the suicide rate of their country of origin [11, 13]. Another possible explanation is related to age composition; immigrants in Norway comprise a relatively younger population than that of the natives [31], and the suicide rate is generally low in populations of young ages [32].

In line with the above notion, caution is needed when interpreting the low suicide mortality of second-generation immigrants. They represent a relatively younger group of people in the Norwegian society, mainly for two reasons. Firstly, many years ago there were quite few people immigrating to Norway; the immigrant population started to increase from the end of 1960s [33]. Second, the identification of second-generation immigrants may be incomplete. In Norway, every live birth receives a personal identifier and is registered with links to their parents at the time of birth. However, for many people born before 1960 links to their parents were not established during the implementation of the Central Population Register [21], so some second-generation immigrants in particular may not be classified as such. Similar selection mechanisms were observed and described in Denmark [34, 35].

Contrary to the first-generation immigrants’ pattern, foreign-born persons with at least one Norwegian-born parent showed a higher suicide rate than native Norwegians. Our data source does not allow us to study more closely characteristics of this subgroup of immigrants. Cases among this population segment might represent intercountry adoptees, which stand out as a high-risk group for psychotic disorders [36] and suicide death [37, 38].

### Suicide methods

This study showed that hanging was the most frequently used suicide method in both native Norwegians and persons with an immigration background, with the highest usage of this method for suicide among first-generation immigrants and foreign-born persons with at least one Norwegian-born parent.

We have previously reported that suicide by hanging in Norway continuously increased between 1969–2012 [39]–a similar trend was also seen in other countries such as Australia and South Korea [40, 41]. Kõlves et *al*. [42] suggested that in Australia, hanging seems to have become more socially acceptable, and a physically and cognitively available method, likely to be linked to low impulse control and short-term suicidal crises. While we are unsure to what extent this notion is applicable in the Norwegian society, the easy availability of hanging materials has no doubt made it a feasible choice by both natives and immigrants as a method to end their own life, especially when other methods such as firearms have been made less accessible in Norway. Additionally, pharmacological agents often used for suicide have gradually become less toxic and/or have been prescribed in smaller quantities and are dispensed as blister packs rather than bottles. Since the end of the 1980s, a stricter firearms legislation and mandatory safe storage of firearms have been implemented in Norway, making firearms less readily available for suicidal individuals, particularly for young males [39, 43]. Still, firearms are likely much more available in the homes of native Norwegians than in the households of first-generation immigrants. Private firearms ownership (around 30% of households [44]) in Norway is nearly exclusively linked to strong hunting traditions in natives still widespread throughout the country. The difference in the use of firearms between natives and first-generation immigrants is more notable in the younger age group (24 years or less) where firearms accounted for almost 30% of all native Norwegian suicides and less than 5% of all first-generation immigrant suicides.

### Suicide occurrence by month of the year

The analysis by the month of the year that suicides occurred showed a peak of suicides in May among the majority population, as well as among first generation immigrants. This is in accordance with a study from Norway on the general population [45]. Many other studies have also shown a spring peak in the occurrence of suicides [46–49], and several explanations to this seasonality have been proposed. The leading theory is the bioclimatic explanation: increased sunshine, long photoperiod, tree pollen, and the influence of other meteorological variables, such as outdoor air temperature, may induce adverse changes in hormones such as melatonin, cortisol, serotonin, and L tryptophan, which play key roles in emotion regulation. Although the exact mechanism is unknown, it is believed that these disturbances may induce dysfunctional emotion regulation which increases the risk of mental ill-health and suicide [50]. Another possible explanation to the suicide peak in spring could be find in the “broken promise” hypothesis, which suggests that suicide is triggered in vulnerable persons when their expectations of better times following winter are unfulfilled [51, 52].

Interestingly, among first-generation immigrants we found a peak in the distribution of suicide also in November. One possible explanation could be that November is the month when the dark and cold weather begins, with low temperatures and less sunlight. In two neighbouring Nordic countries, Finland and Sweden, the monthly suicide peak in the autumn occurred in October and the authors suggested that this suicide peak fully coincided with the biggest annual drop in the seasonal temperature [53]. Immigrants coming from totally different environments may struggle with these hard climate changes and may be negatively affected both physiologically and psychosocially. Immigrants with poor social networks may feel even more socially isolated during such a season while the native Norwegians are likely to spend their time indoors with family and close friends.

### Suicide by time since immigration

This study indicates that over one-fourth of first-generation immigrant suicides occurred during the first five years since immigration, and the proportion was much higher for those from East Europe and Asia, suggesting that the first few years are a crucial time for settlement for these immigrants. Several explanations for this are possible. Recently arrived immigrants usually have fewer social support systems available and less knowledge about available health services for use in times of crisis, and language and cultural barriers may prevent them from effectively seeking help. A review of immigrant mental health research in Norway [54] concluded that immigrants from low-income countries have a higher burden and greater risk for mental health problems than native Norwegians, and that this risk is primarily associated with negative life events both at pre- and post-immigration periods and lack of social support [55–58]. These are all factors that could reduce immigrant resilience and increase the risk of suicide.

Interestingly, our analysis demonstrated that male first-generation immigrants, in particular those aged 35 years or less when they arrived in Norway, tended to die by suicide after fewer years of residence than female first-generation immigrants. Data from Statistics Norway indicate that male first-generation immigrants move to Norway mostly to seek employment, whereas female first-generation immigrants often come to this country for the purpose of family reunification [59]. People who arrive for family reunification purposes might have better access to social support since their family may already be well established in the country [60]. Therefore, females might have a relatively smoother settlement in the country compared to their male counterparts.

Moreover, we found that first-generation immigrants from Eastern Europe, Asia and Africa, aged 35 years or less when moving to Norway, tended to die by suicide years earlier than immigrants from Western Europe. Research from Norway [61] showed that non-Western immigrants had higher levels of psychosocial distress than immigrants from Western countries, and more specifically, in men from non-Western countries, the level of psychosocial distress was highest among those who had arrived in Norway most recently. Immigrants coming from countries culturally distant from Norway (non-Western countries) were less integrated than those from countries culturally closer to Norway; and this integration difference is likely to be more evident in the short period after immigration [61]. Lack of paid employment and the awareness that they failed expectations about being occupationally and financially successful may elevate the risk of suicidal behaviour [9]. Higher unemployment rates among immigrants from Eastern Europe, Asia and Africa compared with immigrants from Western Europe have been reported by Statistics Norway [62].

### Limitations and strengths

Some limitations should be considered when interpreting the findings of this study. First, variables included in the study largely depend on the availability of the data in the source databases. For instance, we were unable to consider reason for immigration (e.g., work, family, refuge, education), mental illness and history of suicide attempts, variables which were not included in our data source for this study and which could provide further insights in suicide characteristics. Secondly, the Central Population Register often does not have records of links to parents for people born before 1960 [21], and this selection mechanism may affect in particular the classification of second-generation immigrants. Moreover, the study was descriptive in its design, and the delineation of the complex relationships among risk factors was beyond the scope of this study.

Among the strengths of this study is the use of national longitudinal registers where data were collected systematically and uniformly, covering all suicides in the whole country over a delineated time period. The quality of data within Norwegian registers has been found to be high [63, 64] due to sound supports from administrative systems where errors are corrected on a continuous and retrospective basis [65]. In particular the degree of coverage and completeness of data in the Cause-of-Death Register is very high; and the diagnoses on the death certificate (reported by doctors) are examined and controlled ensuring they are plausible for a person of the specified age or sex [66]. The use of register-based data also eliminates types of bias such as the recall bias often associated with obtaining data via interviews. In addition, the large number of suicides included in this study yielded good statistical power for estimating differences between natives and various immigrant populations.

### Implications

Detailed information regarding characteristics of suicides in segments of populations in continuing increase is of fundament importance when planning health policies and prevention measures. Hanging was the most common suicide method in all the populations regardless of immigration background, emerging as an important public health concern and a big challenge for suicide prevention. Prevention of suicide by hanging is very complex given the easy availability of hanging materials; therefore prevention should rely on strategies for more effective identification of at-risk individuals and early intervention. Moreover it is important to avoid media coverage on suicide by hanging since this could lead to increased use of this method. Scientific investigations on the impact of media on suicide concluded that media reporting of suicide can lead to imitative suicidal behaviours [67].

Furthermore, the results from this study underscore the importance of considering immigrants as a very heterogeneous group, as differences in characteristics of suicide by immigration background were observed. We observed that first-generation immigrants from non-Western countries tend to die by suicide after fewer years of residence than immigrants from Western countries. This may be indicative of more difficulties in the acculturation processes for immigrants coming from countries culturally distant from Norway (non-Western countries). Lack of social support, poor integration and lack of job opportunities may increase the psychosocial distress in these immigrants, especially in those recently arrived [9, 61]. Thus, suicide prevention among immigrants should rely on measures to improve their social integration, social support and to overcome barriers and difficulties, such as language difficulties and lack of knowledge about available mental health services, which especially most recent immigrants may face when moving to this country [5, 9, 10]. In particular self-management e-resources can play a potentially important role in helping to ensure people get the care and support they need and to reach wide and diverse populations via online resources, social media, and smartphone applications [68]. Immigrants in Norway are less likely than the natives to use services for mental health problems [69], possibly due to different cultural understandings of mental health and the fear of stigma associated with having mental illness. Therefore, offering an alternative to maintain anonymity, e-mental health services may improve the health care accessibility for individuals with immigration backgrounds. Furthermore, anti-stigma initiatives and public information campaigns might have important role in reducing stigma and facilitating contact between immigrants and mental health services [70]; and the school sector may represent an important arena for the implementation of these strategies [71]. Formalized education and training of mental health practitioners in the assessment and treatment of transcultural issues, with the importance of the clinician’ understanding of the patients’ culture of origin, may play a key role in the treatment of immigrants [72].
